# Oncogenic KRAS sensitises colorectal tumour cells to chemotherapy by p53-dependent induction of Noxa

**DOI:** 10.1038/sj.bjc.6605633

**Published:** 2010-03-30

**Authors:** M T de Bruijn, D A E Raats, F J H Hoogwater, W J van Houdt, K Cameron, J P Medema, I H M Borel Rinkes, O Kranenburg

**Affiliations:** 1 Department of Surgery, University Medical Center Utrecht, Heidelberglaan 100, 3584CX Utrecht, The Netherlands; 2 Laboratory of Experimental Oncology and Radiobiology, Center for Experimental Molecular Medicine, Academic Medical Center, Meibergdreef 9, 1105 AZ Amsterdam, The Netherlands

**Keywords:** KRAS, p53, Noxa, oxaliplatin, 5-fluorouracil, colorectal

## Abstract

**Background:**

Oxaliplatin and 5-fluorouracil (5-FU) currently form the backbone of conservative treatment in patients with metastatic colorectal cancer. Tumour responses to these agents are highly variable, but the underlying mechanisms are poorly understood. Our previous results have indicated that oncogenic KRAS in colorectal tumour cells sensitises these cells to chemotherapy.

**Methods:**

FACS analysis was used to determine cell-cycle distribution and the percentage of apoptotic and mitotic cells. A multiplexed RT–PCR assay was used to identify KRAS-controlled apoptosis regulators after exposure to 5-FU or oxaliplatin. Lentiviral expression of short-hairpin RNAs was used to suppress p53 or Noxa.

**Results:**

Oncogenic KRAS sensitised colorectal tumour cells to oxaliplatin and 5-FU in a p53-dependent manner and promoted p53 phosphorylation at Ser37 and Ser392, without affecting p53 stabilisation, p21 induction, or cell-cycle arrest. Chemotherapy-induced expression of the p53 target gene Noxa was selectively enhanced by oncogenic KRAS. Suppression of Noxa did not affect p21 induction or cell-cycle arrest, but reduced KRAS/p53-dependent apoptosis after exposure to chemotherapy *in vitro* and in tumour xenografts. Noxa suppression did not affect tumour growth *per se*, but strongly reduced the response of these tumours to chemotherapy.

**Conclusion:**

Oncogenic KRAS determines the cellular response to p53 activation by oxaliplatin or 5-FU, by facilitating apoptosis induction through Noxa.

Chemotherapy of colorectal cancer mainly involves the fluoropyrimidine drug 5-fluorouracil (5-FU) and the platinum drug oxaliplatin (Chau and Cunningham, 2009). The response of colorectal tumours to these compounds is determined both by the genetic background of tumour cells as well as by environmental cues. Colorectal tumours frequently harbour activating mutations in the *KRAS* proto-oncogene, which drives tumour progression (Andreyev *et al*, 1998, 2001). Signalling by constitutively active Ras proteins can either promote or prevent the induction of apoptosis, depending on the cellular context and on the specific isoform studied (Vos *et al*, 2000; Khokhlatchev *et al*, 2002). For instance, the expression of oncogenic N-Ras but not that of oncogenic K-Ras in the colonic epithelium provides protection against enterocyte apoptosis in a colitis model (Haigis *et al*, 2008). In contrast, we along with others have previously shown that oncogenic KRAS promotes apoptosis of human colorectal tumour cells exposed to either 5-FU or oxaliplatin (Klampfer *et al*, 2005; Smakman *et al*, 2006). Although activating mutations in KRAS alone do not reliably predict the response of colorectal tumours to chemotherapy (Loriot *et al*, 2009), these findings do suggest that the acquisition of KRAS mutations may be associated with an increased propensity to undergo apoptosis. Interestingly, several studies have connected Ras signalling to the activation of p53 (Ries *et al*, 2000; Lin and Lowe, 2001; Ferbeyre *et al*, 2002). In non-transformed cells, the activation of p53 by oncogenic Ras induces a permanent cell-cycle exit and induction of cellular senescence (Serrano *et al*, 1997; Bardeesy and Sharpless, 2006; Ruiz *et al*, 2008). However, p53 activation in (colorectal) tumour cells does not lead to senescence, but rather leads to apoptosis or cell-cycle arrest (Aneja *et al*, 2007; Toscano *et al*, 2007; Braun *et al*, 2008). The tumour suppressor protein p53 is a key player in determining the response of colorectal tumour cells to oncogenic stress and chemotherapy by oxaliplatin and 5-FU (Vousden and Lu, 2002; Adamsen *et al*, 2007; Borralho *et al*, 2007; Toscano *et al*, 2007). The determinants specifying p53 signalling output are the subject of intense investigation. Although p53-induced activation of the CDK inhibitor p21 is sufficient to cause cell-cycle arrest in most cell types, p53-induced apoptosis is more complex and involves the activation of different sets of target genes in different tissues and in response to different stimuli (Oren, 2003; Yu and Zhang, 2005; Kuribayashi and El-Deiry, 2008; Vousden and Prives, 2009). In colorectal tumour cells, the p53-upregulated modulator of apoptosis (PUMA) has an important role in the induction of apoptosis exposed to 5-FU or oxaliplatin (Wang *et al*, 2007). The p53-upregulated modulator of apoptosis belongs to the family of ‘BH3-only’ proteins that promote apoptosis by neutralising Bcl-2 family pro-survival proteins, including Bcl-2, Bcl-x_L_, and myeloid cell leukaemia sequence 1 (Mcl-1) (Willis and Adams, 2005; Shibue and Taniguchi, 2006). p53 also promotes the expression of Noxa, another BH3-only family member (Oda *et al*, 2000). The p53-upregulated modulator of apoptosis is generally considered to be a more potent apoptosis inducer than Noxa, because it can neutralise multiple Bcl-2 family members, whereas Noxa selectively neutralises Mcl-1 (Willis and Adams, 2005; Shibue and Taniguchi, 2006; Ploner *et al*, 2008). However, Noxa-dependent cell death may be particularly important in malignant cells (Suzuki *et al*, 2009), and Noxa could therefore represent a potential therapeutic target.

In this report, we studied the contribution of p53 and p53 target genes to KRAS-induced sensitisation of colorectal tumour cells to 5-FU and oxaliplatin. We found that the mutant KRAS cooperates with p53 in the induction of Noxa, but not other pro-apoptotic p53 target genes. Furthermore, we show that Noxa does not control tumour growth *per se*, but is an important determinant of the tumour response to chemotherapy.

## Materials and methods

### Cell culture

The human colorectal cancer cell line HCT116^G13D/wt^ and the isogenic cell line Hkh2^ko/wt^ lacking the KRAS^D13^ allele were kindly provided by Shirasawa *et al* (1993). HCT116 p53KO cells were kindly provided by Dr Vogelstein. All cells were cultured in Dulbecco's modified Eagle's medium (Dulbecco, ICN Pharmaceuticals, Costa Mesa, CA, USA) supplemented with 5% (v/v) fetal calf serum, 2 mM glutamine, 0.1 mg ml^−1^ streptomycin, and 100 U ml^−1^ penicillin. All cells were kept at 37°C in a humidified atmosphere containing 5% CO_2_. Oxaliplatin was obtained from Sanofi Aventis (Gouda, The Netherlands) and 5-FU was from TEVA (Haarlett, The Netherlands).

### Antibodies

The following antibodies were obtained from Cell Signaling Technology Inc. (Danvers, MA, USA): anti-Puma (no. 4976), anti-caspase-8 1C12 (no. 9746), anti-cleaved caspase-3 ASP175 (no. 9661), and the secondary antibody peroxidase-conjugated anti-rabbit IgG. The following antibodies were all obtained from Santa Cruz Biotechnology (Heidelberg, Germany): anti-p53 DO-1 (sc-126) and anti-p21 C19 (sc-397). Anti-Noxa (IMG-349A) was purchased from Imgenex Corporation (San Diego, CA, USA); anti-*α* tubulin from Sigma Aldrich (St Louis, MO, USA); anti-*β*-actin AC-15 (NB600-501) from Novus Biologicals LLC (Littleton, CA, USA); secondary antibody peroxidase-conjugated anti-mouse IgG from Dako (Glostrup, Denmark); anti-phospho-histone H3 (Ser10) (06-570) from Millipore (Billerica, MA, USA); and the secondary antibody Cy5 AffiniPure F(ab′)_2_ Frag Donkey anti-rabbit IgG from Jackson Immunoresearch Europe (Suffolk, UK). For immunohistochemical staining, anti-Ki-67 clone SP-6 (no. RM-9106S) was acquired from Lab Vision Products Thermo Fisher Scientific (Chesire, UK) and anti-cleaved caspase-3 (no. 559565) from BD Pharmingen (Franklin Lakes, NJ, USA) was used.

### Lentiviral constructs and transduction

The lentiviral short-hairpin RNA (shRNA) construct targeting p53 was kindly provided by Dr AG Jochemsen. Noxa-targeting lentiviral constructs were obtained from the TRC-Mm1.0 library (Sigma Aldrich). The target set used for Noxa (NM_021127) included TRCN0000150555, TRCN0000155978, TRCN0000151311, TRCN0000153637, and TRCN0000155570. Of these constructs, subsequent transduction of cells with TRCN0000151311, followed by transduction with TRCN0000153637 produced the best knock down. As the control vector, we used the same vector containing a sequence targeting luciferase, TGACCAGGCATTCACAGAAAT. For virus production, we used the third-generation packaging system, kindly provided by Professor D Trono.

### p53 phosphorylation status

The phosphorylation status of p53 was analysed using the phospho-p53 antibody sampler kit no. 9919 (Cell Signaling Technology Inc.).

### FACS analysis

Cells were fixed in 70% ice-cold ethanol and incubated for 2 h at 4°C. To determine the percentage of mitosis, cells were washed in PBS-Tween (0.1%) and incubated with anti-phospho-histone H3 antibody for 1 h at RT, after which cells were incubated with CY5-labelled secondary antibody for 1 h at RT. To assess the cell-cycle profile, fixed cells were treated with RNAse and DNA was stained with propidium iodide (PI). All samples were analysed by bivariate flow cytometry using Cell Quest and Modfit software (Becton Dickinson, Breda, The Netherlands).

### MLPA

The MLPA was performed according to the manufacturer's protocol (MRC-Holland BV, Amsterdam, The Netherlands).

### RT–PCR

The RT–PCR was performed by isolating RNA using Trizol, and conversion to cDNA using Superscript2 (Invitrogen, Breda, The Netherlands). The primers used were for Noxa forward: 5′-GGTACCCTGGGAAGAAGGCGCG-3′ and reverse: 5′-GAATTCTCAGGTTCCTGCGCAGAAG-3′. L32 served as a loading control with the forward primer: 5′-GCCCAAGATCGTCAAAAAGA-3′, and reverse 5′-ATTGTGGACCAGGAACTTGC-3′.

### Immunofluorescence

Immunofluoresence was performed as described in the study by Lindqvist *et al* (2007). Images were acquired on a Zeiss LSM510 META microscope (Zeiss, Sliedrecht, The Netherlands). MitoTracker (Invitrogen) was used according to the manufacturer's protocol.

### Tumour model and chemotherapy

All experiments were conducted in accordance with the guidelines of the Animal Welfare Committee of the University Medical Center Utrecht, The Netherlands. Male Balb/C Nu/Nu mice (10–12 weeks) were purchased from Charles River (Sulzfeld, Germany) and were housed in filter top cages. Tumour cells were injected subcutaneously (10^6^ cells in 100 *μ*l 1/3 diluted Matrigel (Becton Dickinson)). When tumours reached ∼100 mm^3^, mice were treated with 12.5 mg kg^−1^ oxaliplatin or vehicle intraperitoneally (day 0). A second dose of oxaliplatin (12.5 mg kg^−1^) was administered on day 7. Tumour growth was assessed every 2 days, and mice were terminated 14 days after the start of treatment. The volume (*V*) was calculated by *V*=*A* × *B*^2^ × 0.5236 (*A*=largest diameter, *B*=diameter perpendicular to *A*).

## Results

### KRAS^D13^ sensitises tumour cells to chemotherapy-induced apoptosis without overriding cell-cycle arrest

**Figure 1:**
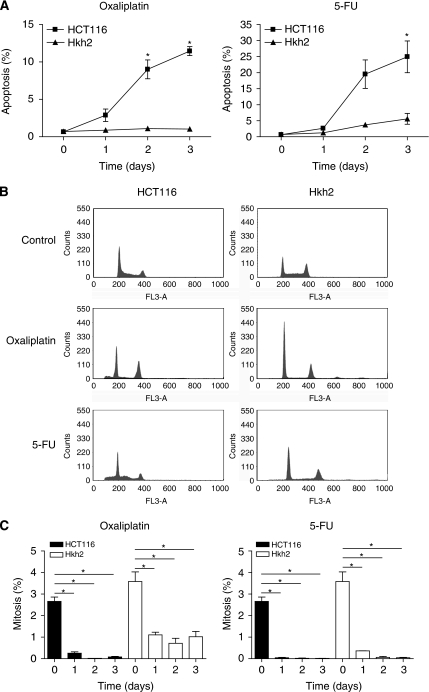
KRAS^D13^ sensitises tumour cells to chemotherapy-induced apoptosis without overriding cell-cycle arrest. (**A**) HCT116 and Hkh2 cells were treated with 8 *μ*g ml^−1^ oxaliplatin or 8 *μ*g ml^−1^ 5-FU for three consecutive days. Cells were then fixed in formalin and stained with propidium iodide (PI). The sub-G1 fraction was determined by FACS analysis. Error bars represent s.e.m. based on three independent experiments (^*^P<0.05). (**B**) Cells were treated as described in panel A. After 2 days, cells were fixed and the cycle profiles of PI-stained cells were analysed by FACS. (**C**) Cells were treated as described in panel A and fixed at the indicated time points. Mitotic cells were then stained using anti-phospho-histone H3 and analysed by FACS. Error bars represent s.e.m., based on three independent experiments (^*^P<0.001).

Oncogenic KRAS may sensitise tumour cells to apoptosis induction by forcing cell-cycle progression in the presence of DNA damage. Therefore, we analysed whether KRAS-facilitated apoptosis induction by 5-FU and oxaliplatin was accompanied by escape from cell-cycle arrest. HCT116 (KRAS^wt/D13^) and isogenic Hkh2 cells (KRAS^wt/ko^) were treated with oxaliplatin and 5-FU for three consecutive days. Apoptosis induction and cell-cycle distribution were then measured by FACS analysis of PI-stained cells. In addition, mitotic progression was measured by FACS analysis of phospho-histone H3-positive cells. We found that KRAS greatly facilitated apoptosis induction by both chemotherapeutics as described previously (Klampfer *et al*, 2005; Smakman *et al*, 2006) (Figure 1A and B). HCT116 cells underwent a *bona fide* cell-cycle arrest and did not enter mitosis before apoptosis induction (Figure 1C). This suggests that KRAS does not promote chemotherapy-induced apoptosis by forcing mitotic entry in the presence of DNA damage.

### KRAS^D13^ promotes apoptosis without affecting p53 stabilisation or p21 induction

**Figure 2:**
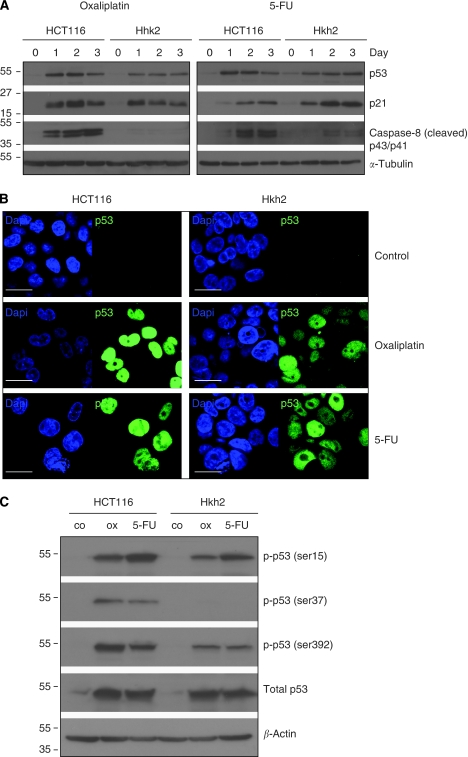
Oncogenic KRAS promotes apoptosis without affecting p53 stabilisation or p21 induction. (**A**) HCT116 and Hkh2 cells were treated with oxaliplatin (8 *μ*g ml^−1^) or 5-FU (8 *μ*g ml^−1^) for 0–3 days as indicated. Lysates were prepared and analysed for the presence of p53, p21, cleaved caspase-8, tubulin, and actin by western blotting. (**B**) HCT116 and Hkh2 cells were treated for 24 h with oxaliplatin or 5-FU. Cells were then processed for immunofluorescence analysis of p53 localisation. Bars represent 50 *μ*m. (**C**) Cells were treated as described in panel B and cell lysates were analysed for the presence of p53 (total p53) and p53 phosphorylated at residues Ser6, Ser9, Ser15, Ser20, Ser37, Ser46, and Ser392. The signals for Ser6, Ser9, Ser20, and Ser46 were below the detection limit.

Next, we tested whether the KRAS status would influence p53 stabilisation. We found that p53 was stabilised to a similar extent in both HCT116 and Hkh2 cells in response to either oxaliplatin or 5-FU (Figure 2A). Nuclear accumulation of p53 was also similar in both cell types (Figure 2B). We were unable to detect p53 localisation to the mitochondria in oxaliplatin- or 5-FU-treated HCT116 or Hkh2 cells (Supplementary Figure 1). However, whereas p53 stabilisation correlated caspase-8 cleavage in HCT116 cells, this was not observed in Hkh2 cells (Figure 2A). Induction of the cyclin-dependent kinase inhibitor and p53 target p21 by chemotherapy was not affected by KRAS status (Figure 2A), which is in line with the finding that both apoptosis-prone and apoptosis-resistant cells undergo cell-cycle arrest.

### KRAS promotes p53 phosphorylation of p53 on Ser37 and Ser392

The differential regulation of p53 target genes is controlled by both post-translational modification and its binding partners. In this study, we tested whether KRAS status modified chemotherapy-induced phosphorylation of p53. We found that oxaliplatin- or 5-FU-induced p53 phosphorylation on Ser15 was not altered, but phosphorylation on Ser37 and Ser392 was far more pronounced in cells expressing oncogenic KRAS (Figure 2C). Again, p53 stabilisation was not different between HCT116 and Hkh2 cells (Figure 2C).

### p53 is required for KRAS^D13^-stimulated tumour cell sensitisation to apoptosis

**Figure 3:**
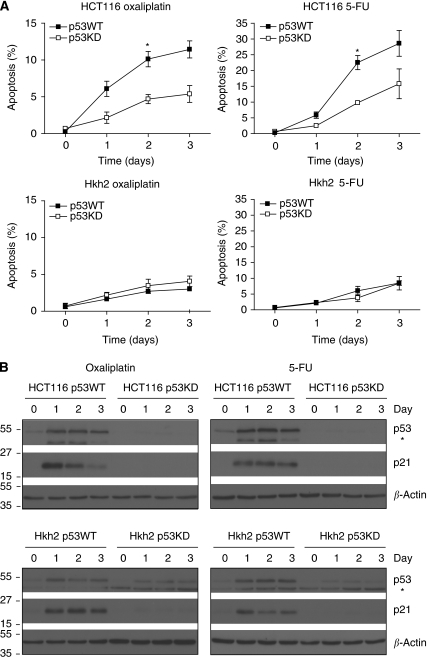
Wild-type p53 is required for sensitisation to apoptosis by oncogenic KRAS. (**A**) HCT116 and Hkh2 cells were transduced with a lentiviral control shRNA vector, and with a vector targeting p53. All four cell lines were then treated for 0–3 days with oxaliplatin (8 *μ*g ml^−1^) or 5-FU (8 *μ*g ml^−1^). After fixation, cells were stained with propidium iodide and the sub-G1 fraction was determined by FACS analysis. Error bars represent s.e.m. of three independent experiments (^*^P<0.05). (**B**) Cells were treated as described in panel A, and cell lysates were prepared and analysed for the presence of p53 and p21 by western blotting.

Next, we tested whether p53 was required for the sensitising effect of KRAS^D13^ on chemotherapy-induced tumour cell apoptosis. To this end, we created stable p53 knockdown cell lines by using lentiviral RNA interference. Knockdown of p53 in HCT116 cells strongly reduced apoptosis in response to oxaliplatin or 5-FU in HCT116 cells (Figure 3A, upper panel). However, p53 knockdown did not affect 5-FU- or oxaliplatin-induced apoptosis in Hkh2 cells (Figure 3A, lower panel). In both cell types, p21 induction was completely abrogated (Figure 3B). These results show that the apoptosis-promoting effect of KRAS^D13^ depends on the presence of wild-type p53.

### 5-FU and oxaliplatin induction of Noxa requires oncogenic KRAS and wild-type p53

**Figure 4:**
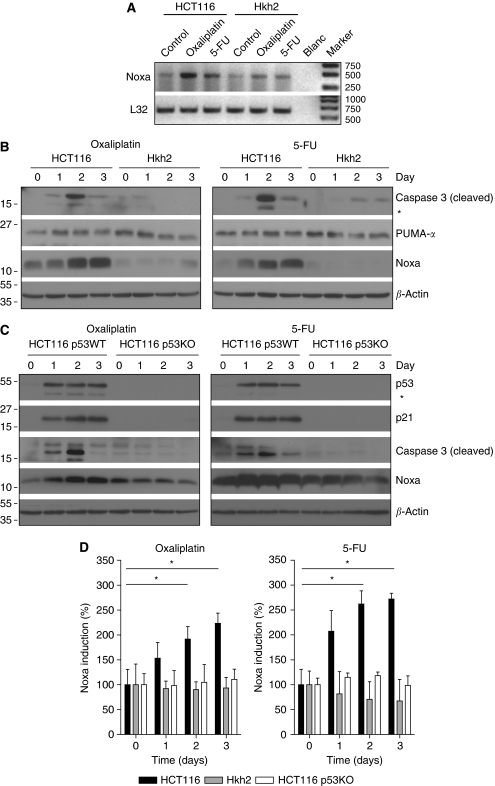
Oncogenic KRAS is required for p53-dependent induction of Noxa by chemotherapy. (**A**) HCT116 and Hkh2 cells were treated with oxaliplatin (8 *μ*g ml^−1^) or 5-FU (8 *μ*g ml^−1^) for 24 h. Total RNA was then isolated and tested for the presence of Noxa and L32 mRNA by RT–PCR. (**B**) Cells were treated for 0–3 days with oxaliplatin or 5-FU, and Noxa protein levels were determined by western blot analysis. (**C**) HCT116 expressing (HCT116 p53WT) or lacking p53 (HCT116 p53KO) were cultured in the presence of oxaliplatin or 5-FU for 0–3 days as indicated. Lysates were prepared and analysed for the presence of p53, p21, PUMA, and Noxa protein levels. (**D**) The experiment described in panel C was repeated three times and all Noxa blots were scanned densitometrically to quantify the induction. Graphs represent % induction relative to day 0. ^*^Indicates statistically significant differences (*P*<0.05).

To gain further insight into the mechanism by which oncogenic KRAS facilitates apoptosis induction in response to chemotherapy, we performed a multiplexed RT–PCR analysis (MLPA) for the induction of apoptosis control genes by either 5-FU or oxaliplatin in HCT116 and Hkh2 cells (Supplementary Figure 2A). Of the 32 genes tested in this screen, only Noxa (PMAIP1) was selectively induced in HCT116 cells. The Noxa-related PUMA gene was induced to a similar extent in HCT116 and Hkh2 cells by 5-FU or oxaliplatin. However, chemotherapy induction of PUMA mRNA levels was relatively low. The PUMA/Noxa binding partner Mcl-1 was not induced by either drug (Supplementary Figure 2B). Both RT–PCR and western blot analysis confirmed the selective induction of Noxa in HCT116 cells, but not in Hkh2 cells (Figure 4A and B) and the relatively poor induction of PUMA (Figure 4B). Thus, induction of the p53 target gene Noxa by 5-FU and oxaliplatin requires the presence of oncogenic KRAS. Next, we tested the dependency of Noxa induction on p53 in HCT116 cells. As expected, the induction of Noxa by oxaliplatin or 5-FU was strongly reduced in cells lacking p53 (Figure 4C). Quantification of Noxa induction in response to chemotherapy by densitometric scanning of the blots of three independent experiments showed that Noxa induction requires the presence of both wild-type p53 and oncogenic KRAS (Figure 4D).

### A critical role for Noxa in apoptosis induction by oxaliplatin and 5-FU

**Figure 5:**
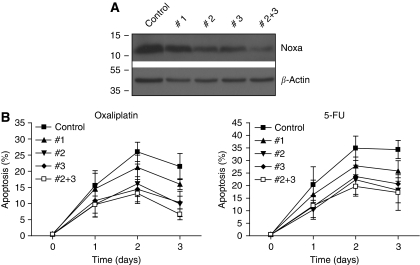
Contribution of Noxa to KRAS-facilitated apoptosis induction in response to chemotherapy. (**A**) HCT116 cells were transduced with lentiviral vectors targeting Noxa (no. 1, no. 2, no. 3, and no. 2+no. 3), or with a vector targeting firefly luciferase (control). After selection in puromycin-containing medium for 1 week, cells were lysed and analysed for Noxa protein levels by western blot analysis. (**B**) HCT116 control and Noxa knockdown (NoxaKD) cells were treated with oxaliplatin (8 *μ*g ml^−1^) or 5-FU (8 *μ*g ml^−1^) for 0–3 days. The percentage of apoptotic cells was then determined by FACS analysis as in Figure 1. Error bars represent s.e.m. of three independent experiments.

We next tested whether KRAS-dependent induction of Noxa was instrumental in sensitising HCT116 cells to oxaliplatin and 5-FU. To this end, we created stable cell lines in which Noxa was suppressed by RNA interference. Of a set of five Noxa-targeting shRNA constructs, two (nos 2 and 3) reduced Noxa protein levels to ∼30% of control levels. Combination of these targeting constructs further reduced Noxa levels to ∼10% of control levels (Figure 5A). Exposure of control cells and Noxa-suppressed cells to either oxaliplatin or 5-FU showed that Noxa suppression protected HCT116 cells from apoptosis induction by 50–70% over a period of 3 days. In contrast, the relatively inefficient targeting construct no. 1 (20–30% knockdown) had only a marginal protective effect (Figure 5A and B), when compared with control cells expressing luciferase-targeting shRNA.

### Therapeutic efficacy of oxaliplatin and induction of tumour cell apoptosis requires Noxa

**Figure 6:**
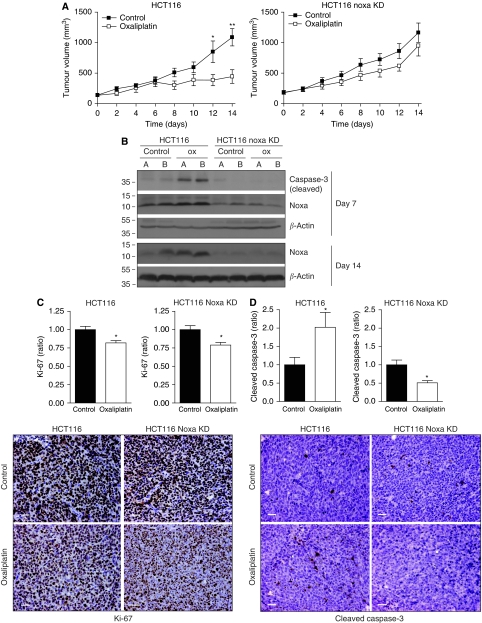
Contribution of Noxa to the therapeutic efficacy of oxaliplatin and 5-FU. (**A**) Immune-deficient nude mice (*n*=8 per group) were injected subcutaneously with 10^6^ HCT116 control or HCT116-NoxaKD cells. When tumours reached a size of 100 mm^3^, the mice received either PBS or oxaliplatin (12.5 mg kg^−1^) intraperitoneally (day 0). Seven days later, all mice received a second dose of either PBS or oxaliplatin. Tumour growth was followed by caliper measurements every 2 days (^*^P=0.048, ^**^P=0.008). (**B**) Tumours were harvested on day 7 and day 14. Of each group, two tumours (panels A, B) were analysed for the presence of cleaved caspase-3 (as a marker for apoptosis) and for Noxa by western blotting. All tumours were analysed by immunohistochemistry for Ki-67 (**C**) and cleaved caspase-3 (**D**) as markers for proliferation and apoptosis respectively. Ten random fields per tumour were scored and the positive tumour areas were determined by automated computer analysis. The bar graphs represent the ratios of Ki-67 and caspase-3-positive surface areas in oxaliplatin-treated xenografts *vs* control xenografts (^*^P<0.05). Representative images are shown on the right. Bars represent 50 *μ*m.

We next tested whether suppression of Noxa affected tumour growth and therapy resistance. To this end, control HCT116 cells (luc-shRNA) and Noxa-suppressed HCT116 cells were injected into the flanks of nude mice and tumour growth was followed over time. When tumours reached a size of 100 mm^3^, PBS or oxaliplatin treatment was initiated. Tumour growth in control mice was unaffected by Noxa suppression (Figure 6A). Oxaliplatin treatment strongly reduced the growth of control HCT116 tumours, but had virtually no effect on Noxa-suppressed tumours (Figure 6A). Tumours were then excised and analysed by western blotting and immunohistochemistry, using Ki-67 and activated caspase-3 as markers for proliferation and apoptosis, respectively. Noxa suppression was stable during the course of the experiment (Figure 6B). Induction of caspase-3 processing by oxaliplatin was completely abolished in Noxa-suppressed tumours (Figure 6B and D). Oxaliplatin had a marginal but statistically significant inhibitory effect on tumour cell proliferation, but this was not affected by Noxa suppression (Figure 6C). These results show that Noxa is a key determinant of the apoptotic, but not the cytostatic response of HCT116 tumours to oxaliplatin chemotherapy.

## Discussion

In this study, we provide evidence that oncogenic KRAS facilitates chemotherapy-induced apoptosis of HCT116 colorectal tumour cells by cooperating with p53 in the induction of the pro-apoptotic gene Noxa. p53-dependent induction of PUMA did not require oncogenic KRAS. Both PUMA and Noxa belong to the class of BH3-only proteins that function as neutralisers of the pro-survival Bcl-2-like proteins (Willis and Adams, 2005). The contribution of PUMA and Noxa to apoptosis induction varies considerably between cell types (Shibue *et al*, 2003, 2006; Villunger *et al*, 2003; Michalak *et al*, 2008). Apoptosis induction in colorectal cancer cells by chemotherapy is partly (∼50%) dependent on the induction of PUMA (Wang *et al*, 2007). Noxa is expressed both in the normal intestine and in most intestinal tumours (Jansson *et al*, 2003), and Noxa deficiency strongly reduces p53-dependent apoptosis in intestinal crypts following *γ*-irradiation (Shibue *et al*, 2003). Our results show that Noxa also has an essential role in chemotherapy-induced KRAS/p53-dependent apoptosis induction in intestinal (colorectal) cancer cells. Taken together, optimal p53-dependent apoptosis in colorectal cancer cells presumably requires induction of both Noxa and PUMA to achieve maximal neutralisation of Bcl-2 pro-survival proteins.

Importantly, p53 is not the only regulator of Noxa gene expression. Transcription factors such as c-Myc, E2F1, and HIF1*α* regulate Noxa expression in a p53-independent manner (Hershko and Ginsberg, 2004; Hershko *et al*, 2005; Nikiforov *et al*, 2007; Ploner *et al*, 2008). Interestingly, oncogenic KRAS is critical for maintaining high c-Myc levels in the HCT116/Hkh2 system (Shirasawa *et al*, 1993), and can promote expression of E2F1 (Berkovich and Ginsberg, 2001) and stabilisation of HIF1*α* (Kikuchi *et al*, 2009). The stimulatory effect of oncogenic KRAS on one or more of these transcription factors may explain why basal Noxa levels are low in KRAS^D13^-deleted tumour cells. p53 is not required for maintaining basal levels of Noxa, but cooperates with oncogenic KRAS to induce Noxa expression in response to chemotherapy.

Expression of the H-Ras oncogene in primary cells causes stabilisation of p53 by induction of the tumour suppressor p19^ARF^ (p14^ARF^ in human cells) (Palmero *et al*, 1998). However, despite the presence of a mutant *KRAS* allele in HCT116 cells, its p14^ARF^ levels are relatively low (Javelaud and Besancon, 2002), and we did not observe overt differences in basal or chemotherapy-induced p53 stabilisation in cells with or without KRAS^D13^. The control of p53 signalling output by oncogenic KRAS may therefore involve alterations in p53 post-translational modifications and/or binding partners. Our study showed that deletion of KRAS^D13^ strongly reduced chemotherapy-induced phosphorylation of p53 at Ser37 and Ser392. Phosphorylation of both residues has been associated with the transcriptional output of p53, although not with apoptosis-specific gene regulation. As KRAS status did not affect p53 stabilisation or p21 induction, KRAS^D13^-controlled phosphorylation of Ser37 and/or Ser392 may contribute to specifying p53 target gene induction. Interestingly, Ser37 phosphorylation augments p53 acetylation by p300 (Sakaguchi *et al*, 1998) and this promotes Noxa induction and apoptosis (Terui *et al*, 2003). Phosphorylation of p53 at Ser46, Ser15, and Ser20 has also been implicated in apoptosis-specific p53 signalling (Aylon and Oren, 2007). However, phosphorylation of these residues was either not detected (Ser20, Ser46) or not regulated by KRAS status (Ser15) in the HCT116 cell system.

Several additional determinants of the tumour cell response to p53 activation have been identified. These include p53 post-translational modifications, interaction partners, and proteins that occupy p53 target gene promoters independently of p53 (Vousden and Prives, 2009). Whether oncogenic KRAS alters any of these additional pathways or whether differential phosphorylation at Ser37 and/or Ser392 is sufficient to skew p53 signalling output towards Noxa induction and apoptosis will be the subject of further studies.

Oxaliplatin and 5-FU are the mainstay chemotherapeutics in the treatment of patients with disseminated colorectal cancer. However, there are currently no reliable predictors for response to chemotherapy (Allen *et al*, 2006; Longley *et al*, 2006; Walther *et al*, 2009). Meta-analyses of the literature on the impact of p53 abnormalities and KRAS mutations on therapy outcome showed that neither p53 nor KRAS status had a predictive value (Munro *et al*, 2005; Loriot *et al*, 2009). Our results suggest that tumours expressing oncogenic KRAS in combination with wild-type p53 may respond best to chemotherapy. To the best of our knowledge, this has so far not been addressed in large patient cohorts. Noxa expression in a cohort of colorectal cancer patients treated with 5-FU was unrelated to treatment response (Sinicrope *et al*, 2008). However, such negative correlations are hard to interpret, given that Noxa induction in response to chemotherapy is transient. Colorectal tumours express Noxa at normal levels (Jansson *et al*, 2003), and inactivating mutations have so far not been reported. Therefore, the loss of Noxa function does not seem to be required during colorectal tumour development. Indeed, the Noxa-dependent apoptosis pathway is intact in colorectal tumour cells (Ravi *et al*, 2004; Okumura *et al*, 2008). Furthermore, Noxa has been identified as a tumour-specific inducer of breast carcinoma cell death that spares non-transformed mammary cells (Suzuki *et al*, 2009). These observations suggest that the Noxa pathway may be an attractive target for therapeutic exploitation.
