# Supplementary material for: Oncogenic KRAS sensitises colorectal tumour cells to chemotherapy by p53-dependent induction of Noxa
Source: Br J Cancer. 2010 Mar 30;102(8):1254–64. doi: 10.1038/sj.bjc.6605633 (PMC2856010; doi:10.1038/sj.bjc.6605633)
Supplement: Supplementary Figure 1 [file 6605633x1.pdf]

Supplementary figure 1

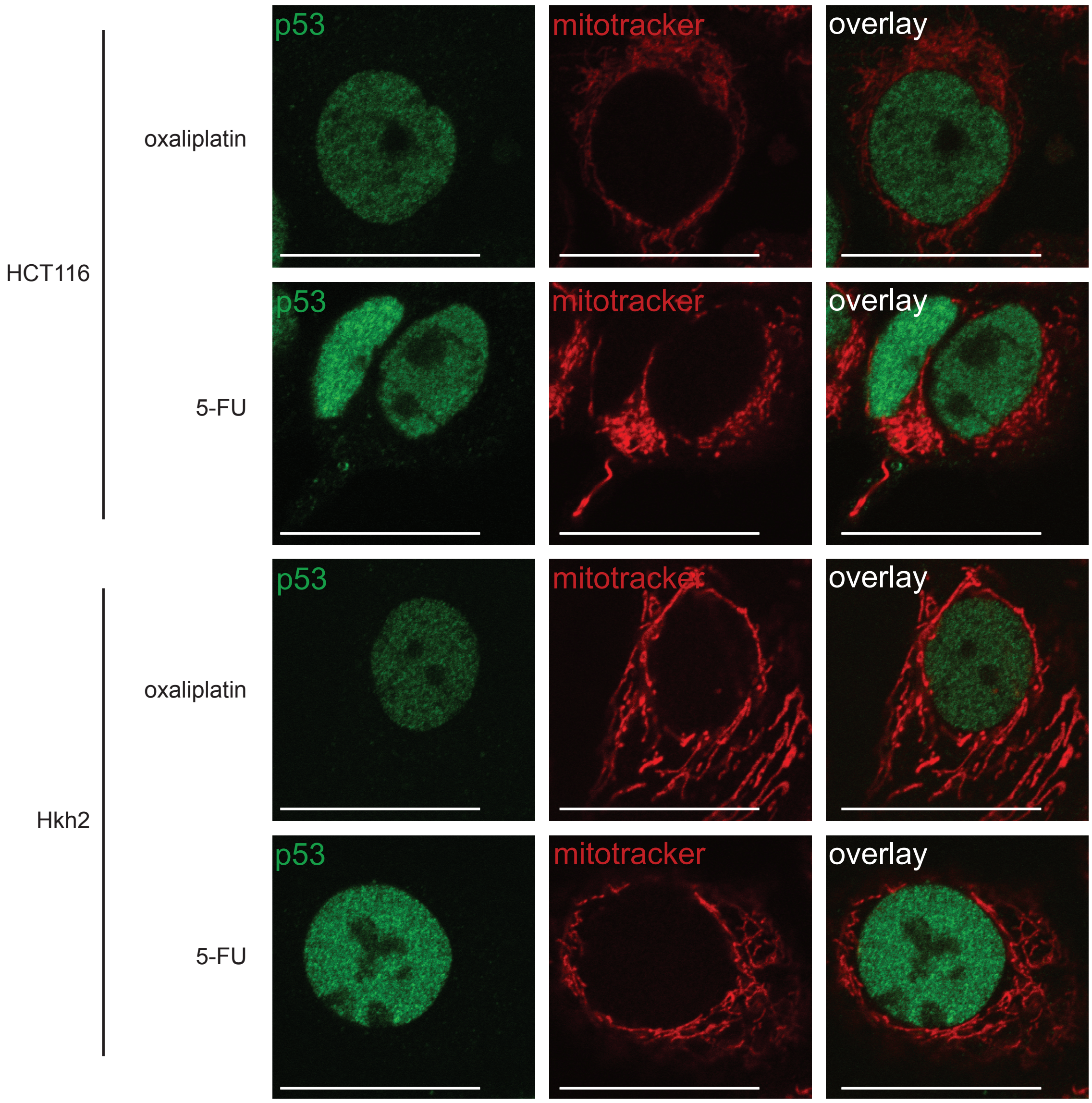

Supplementary figure 1
p53 does not co-localize with mitochondria. Cells were treated for 48 hours with 8µg/ml oxaliplatin or 8µg/ml 5-FU, which was followed by fixation and staining for p53 (green) and mitochondria (red). Bars represent 30µm.
