# Supplementary material for: Oncogenic KRAS sensitises colorectal tumour cells to chemotherapy by p53-dependent induction of Noxa
Source: Br J Cancer. 2010 Mar 30;102(8):1254–64. doi: 10.1038/sj.bjc.6605633 (PMC2856010; doi:10.1038/sj.bjc.6605633)
Supplement: Supplementary Figure 2 [file 6605633x2.pdf]

Supplementary figure 2

A

	Induction					
**Genes**	**HCT116 control**	**HCT116 oxaliplatin**	**HCT116 5-FU**	**HKH2 control**	**HKH2 oxaliplatin**	**HKH2 5-FU**
**Tnfrsf21**	6,43	4,11	2,69	0,57	1,56	1,25
**Cdkn1a**	16,58	76,94	61,15	5,30	23,84	34,18
**Htra2**	2,10	1,47	0,97	0,63	1,09	0,97
**Bcl2L2**	0,42	0,00	0,35	0,13	0,28	0,28
**Cflar v11**	0,36	1,19	0,93	0,25	0,56	0,71
**Bcl2L1**	6,70	8,92	6,91	2,80	5,92	5,51
**PMAIP1**	7,48	32,52	27,59	1,47	3,86	5,47
**Cflar v22**	0,27	0,00	0,00	0,11	0,21	0,00
**MCL1**	3,52	3,71	3,20	1,59	2,36	2,06
**B2M**	1,00	1,00	1,00	1,00	1,00	1,00
**BAD**	1,66	1,29	0,91	0,75	1,02	0,95
**BAX**	5,60	8,94	4,66	1,34	4,54	3,75
**BOK**	2,17	1,68	0,71	1,12	2,16	1,17
**Bcl2L11**	4,54	5,55	5,40	1,68	3,25	3,95
**BID**	3,04	3,38	3,16	1,64	3,27	3,69
**MIL1**	3,16	2,54	1,83	1,46	2,32	1,65
**HRK**	0,00	0,00	0,00	0,35	0,31	0,00
**Birc1**	0,24	0,00	0,00	0,11	0,00	0,00
**Birc3**	0,90	1,35	2,25	0,39	1,99	3,28
**Birc5**	8,96	1,58	0,00	1,91	2,86	1,04
**Birc6**	1,58	1,45	1,41	0,47	1,09	0,88
**SerpinB9**	2,99	1,25	0,97	0,39	0,61	0,38
**Bbc3**	1,59	2,58	3,96	1,03	1,95	2,90
**Parn**	1,48	1,10	0,91	0,50	1,09	0,74
**Pdcd8**	2,96	2,37	1,97	0,73	1,60	1,28
**Moap1**	0,59	0,96	1,07	0,30	0,61	0,66
**Gusb**	1,01	0,00	0,00	0,30	0,32	0,00
**Bnip3L**	1,37	1,06	1,14	0,72	1,12	1,07
**APAF1**	2,06	2,03	2,22	0,37	1,19	0,98
**Diablo**	3,61	2,69	2,57	1,21	2,57	2,13
**Bmf**	0,00	0,00	0,00	0,16	0,00	0,00
**Bik**	0,58	1,13	1,47	0,20	0,42	0,54

B

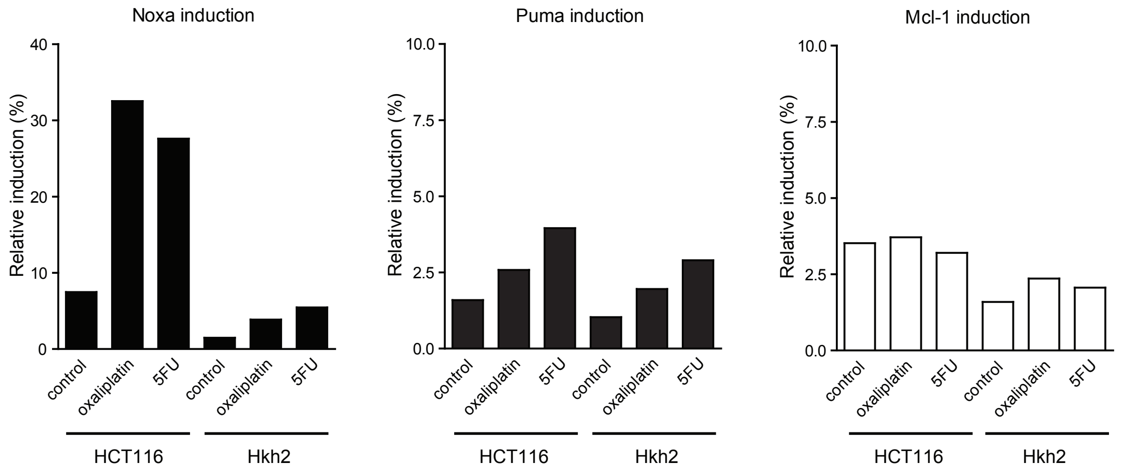

Supplementary figure 2
Multiplex assay for apoptosis related genes. (***A***) HCT116 and Hkh2 cells were treated for 48 hours with 8µg/ml oxaliplatin or 8µg/ml 5-FU, and analyzed according to manufacturers protocol. Induction is relative to housekeeping gene B2M. (***B***) Bar graphs representing individual MLPA data for Noxa and PUMA (p53's main pro-apoptotic targeting genes), with and without correction for the anti-apoptotic binding partner Mcl-1.
